# Decision regret of cancer patients after radiotherapy: results from a cross-sectional observational study at a large tertiary cancer center in Germany

**DOI:** 10.1007/s00432-024-05638-0

**Published:** 2024-03-28

**Authors:** Alexander Rühle, Leonie Wieland, Andreas Hinz, Anja Mehnert-Theuerkauf, Nils H. Nicolay, Clemens Seidel

**Affiliations:** 1 Department of Radiation Oncology, University Medical Center Leipzig, Stephanstr. 9a, 04103 Leipzig, Germany; 2 Comprehensive Cancer Center Central (CCCG) Germany, Partner Site Leipzig, Leipzig, Germany; 3 Department of Medical Psychology and Medical Sociology, University Medical Center Leipzig, Leipzig, Germany

**Keywords:** Radiotherapy, Chemotherapy, Quality of life, Psycho-oncology, Patient-reported outcomes

## Abstract

**Purpose:**

The decision-making process regarding cancer treatment is emotionally challenging for patients and families, harboring the risk of decision regret. We aimed to explore prevalence and determinants of decision regret following radiotherapy.

**Methods:**

This cross-sectional observational study was conducted at a tertiary cancer center to assess decision regret following radiotherapy. The study employed the German version of the Ottawa Decision Regret Scale (DRS) which was validated in the study population. Decision regret was categorized as absent (0 points), mild (1–25 points), and strong (> 25 points). Various psychosocial outcome measures were collected using validated questionnaires to identify factors that may be associated with decision regret.

**Results:**

Out of 320 eligible patients, 212 participated, with 207 completing the DRS. Median age at start of radiotherapy was 64 years [interquartile range (IQR), 56–72], genders were balanced (105 female, 102 male), and the most common cancer types were breast (*n* = 84; 41%), prostate (*n* = 57; 28%), and head-and-neck cancer (*n* = 19; 9%). Radiotherapy was applied with curative intention in 188 patients (91%). Median time between last radiotherapy fraction and questionnaire completion was 23 months (IQR, 1–38). DRS comprehensibility was rated as good or very good by 98% (196 of 201) of patients. Decision regret was reported by 43% (*n* = 90) as absent, 38% (*n* = 78) as mild, and 18% (*n* = 38) as strong. In the multiple regression analysis, poor Eastern Cooperative Oncology Group performance status, low social support, and dissatisfaction with care were independent risk factors for higher decision regret after radiotherapy.

**Conclusions:**

The German version of the DRS could be used to assess decision regret in a diverse cohort of cancer patients undergoing radiotherapy. Decision regret was prevalent in a considerable proportion of patients. Further studies are necessary to validate these findings and obtain causal factors associated with decision regret after radiotherapy.

**Supplementary Information:**

The online version contains supplementary material available at 10.1007/s00432-024-05638-0.

## Introduction

Decision regret is a multifaceted emotional response encompassing feelings of disappointment and remorse concerning the choices made during the treatment decision-making process (Landman 1987). It can be described as a feeling that opting for a different course of action would have resulted in a more favorable outcome in the current situation (Coricelli et al. 2007). This adverse emotional sensation can arise when uncertainty surrounding the optimal choice remains unresolved or when an undesirable outcome prompts the belief that an alternative decision might have been more beneficial (Joseph-Williams et al. 2011). This phenomenon is particularly pertinent in the field of oncology, where treatment decisions often carry profound implications for patients' lives (Connolly and Reb 2005). Prevalence and determinants of decision regret were studied in several cancer types, such as prostate cancer (Wallis et al. 2022), breast cancer (Martinez et al. 2015), lung cancer (Sullivan et al. 2023), and head-and-neck cancer (Nallani et al. 2022). The Ottawa Decision Regret Scale (DRS) was initially developed by Brehaut and colleagues and has demonstrated its validity and reliability as a measurement tool for decision regret (Brehaut et al. 2003).

Radiotherapy is a cornerstone in cancer treatment, and about 50% of all cancer patients in Europe undergo at least one course of radiotherapy during their disease (Lievens et al. 2020). While numerous studies have explored decision regret in various healthcare settings, its prevalence and determinants in the context of radiotherapy remain underexplored (de Groot et al. 2012; Nallani et al. 2022; Windon et al. 2019; Zoumpou et al. 2023). Decision regret for (1) not omitting radiotherapy after surgery (e.g., in older women with low-risk breast cancer (Kunkler et al. 2023)), (2) for not deciding to undergo primary surgery instead of radical radiotherapy [e.g., in men with localized prostate cancer (Hamdy et al. 2023)], (3) for not opting for a different radiotherapy fractionation schedule [e.g., short-course neoadjuvant radiotherapy instead of long-course neoadjuvant chemoradiation in patients with rectal cancer (Ciseł et al. 2019)] or (4) for a less aggressive radiotherapy regimen (e.g., omitting radiotherapy boost to the tumor bed or omitting coverage of the elective lymph nodes in women with breast cancer (Bartelink et al. 2015)) are only some examples that may occur in patients after radiotherapy. Understanding the factors contributing to decision regret after radiotherapy is crucial not only for enhancing patient satisfaction and well-being but also for refining the shared decision-making process between patients and healthcare providers.

Decision regret may be dependent on cultural differences (Hawley and Morris 2017; López et al. 2014; Shaw et al. 2015), providing a rationale to examine this issue separately for individual countries. However, there is a paucity of studies about decision regret after radiotherapy in cancer patients in Germany (Köksal et al. 2023, 2022). We therefore aimed at investigating the prevalence and determinants of decision regret in cancer patients who were treated with radiotherapy for their disease at a large tertiary German cancer center. Previous studies have found relationships between decision regret and various patient-reported outcomes, such as quality of life (Calderon et al. 2019), distress, depression, anxiety (Sheehan et al. 2007), perception of the decision-making process (Yamauchi et al. 2019), satisfaction with care (Berkowitz et al. 2021), social support (Wallis et al. 2022), and health literacy (Joyce et al. 2020), wherefore we also surveyed these patient-reported psychosocial outcome measures to find potential variables that are associated with decision regret. We also aimed to examine the reliability of the German version of the DRS in a broad group of cancer patients, in order to facilitate its usage in subsequent studies.

## Materials and methods

### Study design

This was a cross-sectional observational study performed at the Department of Radiation Oncology, Leipzig University Medical Center. Inclusion criteria for this study were (1) at least one course of radiotherapy for a malignant disease, (2) age of ≥ 18 years, (3) the ability to understand the German questionnaires, and (4) informed consent to participate in this study. Cancer patients who attended follow-up appointments after radiotherapy between July 10, 2023 and August 18, 2023 in the outpatient clinic of the Department of Radiation Oncology, Leipzig University Medical Center, were eligible for the study and were actively asked to participate in the study. Participating patients were able to either fill out the questionnaires during the waiting time for the medical consultation, or at home after the follow-up appointment. The study was approved in advance by the local ethics committee (reference number 077/23-ek) and was performed in accordance with the declaration of Helsinki. All patients provided written informed consent.

### Questionnaires and variables

The German version of the 5-item Ottawa DRS (Brehaut et al. 2003), which has previously been validated concerning decision regret in patient caregivers [DRS-C (Haun et al. 2019)], was used in this study. A forward translation of the original DRS was also performed (A.M.T.) and results were compared with the existing German DRS-C. The German DRS-C and the independently translated DRS were almost identical, so that we decided to use the same wording as the German DRS-C in order to ensure comparability. The German version of the DRS is shown in the Supplementary Material. DRS scores range from 0 to 100, with 0 indicating no regret and 100 indicating a high degree of regret. Following the previous validation study of the Ottawa DRS, degree of decision regret was subdivided into absent (0 points), mild (1–25 points), and strong (> 25 points) decision regret (Brehaut et al. 2003). In addition, patients were asked to indicate the comprehensibility and explicitness of the DRS using 5 questions, each with a 5-point Likert scale (Supplementary Material). For reliability analysis, Cronbach's alpha was calculated to assess the internal consistency. In order to find determinants of decision regret, various patient-reported psychosocial outcome measures were collected. Quality of life was measured with the European Organisation for Research and Treatment of Cancer (EORTC) QLQ-C30 (Aaronson et al. 1993) questionnaire, and distress was assessed with the German version of the National Comprehensive Cancer Network (NCCN) distress thermometer ranging from 0 (no distress) to 10 (extreme distress) (Mehnert et al. 2006). Depression and anxiety were measured using the German versions of the Patient Health Questionnaire-9 (PHQ-9) (Kroenke et al. 2001) and Generalized Anxiety Disorder Screener (GAD-7) (Spitzer et al. 2006), respectively. Patients' experiences concerning participation in the decision-making process of radiation treatment were examined using the Shared Decision Making Questionnaire (SDM-Q-9) (Kriston et al. 2010), and patient satisfaction with care during radiotherapy was quantified with the Satisfaction with Comprehensive Cancer Care (SCCC) questionnaire (Esser et al. 2021). Here, patients were explicitly asked to answer these questionnaires according to their last radiotherapy course. Assessment of social support was performed with the SSUK-8 (Ullrich and Mehnert-Theuerkauf 2010), while health literacy was assessed with the HLS-EU-Q16 (Niedorys et al. 2020). Only paper questionnaires were used in the study. In addition, patient and treatment characteristics regarding the last course of radiotherapy were retrospectively extracted from the medical records. Tumor stage at the time of radiotherapy was indicated based on the current staging classification system at that time. Possible radiotherapy-related toxicities at the time of study participation were classified according to the Common Terminology Criteria for Adverse Events (CTCAE) v5.0.

### Statistical analysis

All statistical analyses were performed using IBM SPSS Statistics version 29 (IBM Corp., Armonk, NY, USA). Descriptive statistics were used to display the study cohort. To investigate the univariate relationships between distress and covariates, Pearson correlations and one-way analysis of variance (ANOVA) tests were employed based on the scale of the respective covariate. If the one-way ANOVA yielded significant results, Tukey’s post hoc testing was applied. For exploring the multivariable connections between decision regret and covariates, a linear regression with decision regret as dependent variable was performed. In the regression analysis, listwise deletion of missing data was performed (i.e., complete-case analysis). Assumptions of a linear regression analysis (linearity, normality, homoscedasticity, absence of multi-collinearity, uncorrelatedness of residuals) were checked. Details of the model performance and the assumption test results are shown in the Supplementary Material. Considering the exploratory nature of our analyses, we did not perform adjustments for multiple testing. A *p* value of < 0.05 was considered statistically significant for all analyses.

## Results

### Study flow diagram

**Fig. 1:**
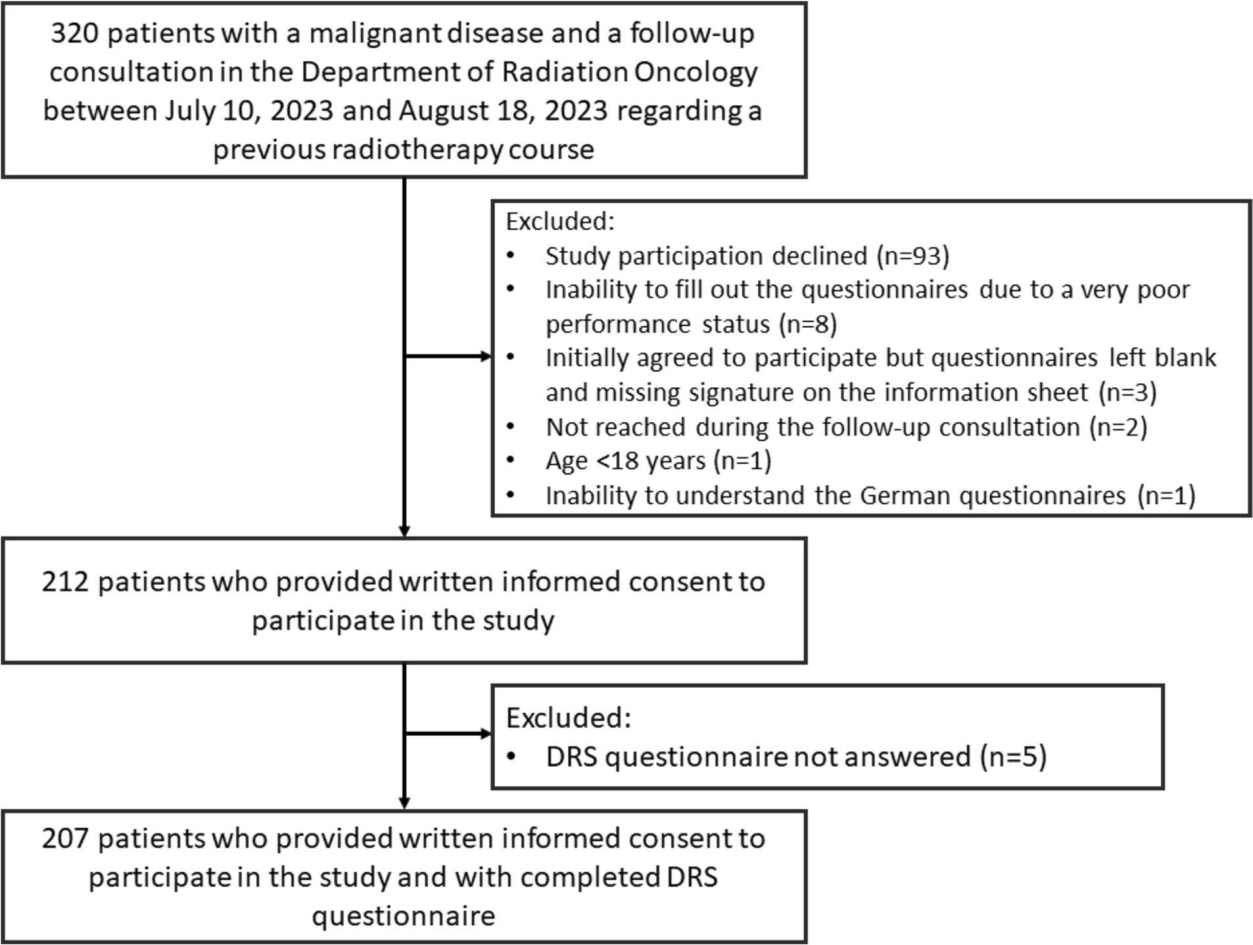
Study flow diagram. DRS, decision regret scale

This cross-sectional study recruited a total of 212 patients out of 320 eligible patients (66% response rate) (Fig. 1). Of these 212 patients, 207 completed the DRS and were included in the final analysis. A comparison between patients who refused to participate in the study and patients who agreed to participate is shown in the Supplementary Table 1. While age, gender, and primary cancer were not significantly different between participants and non-participants, Eastern Cooperative Oncology Group (ECOG) performance status was significantly worse in non-participants (*p* < 0.001, *χ*^2^-test).

### Characteristics of the study cohort

Patient and treatment characteristics are shown in Table 1. The median age of the participants was 64 years (IQR, 56–72), with a balanced distribution of gender (*n* = 105 female, *n* = 102 male). The most prevalent cancer types were breast cancer (*n* = 84; 41%), prostate cancer (*n* = 57; 28%), and head-and-neck cancer (*n* = 19; 9%). Curative radiotherapy (including radiotherapy for oligo-metastatic disease) was administered to 188 patients (91%), while the remaining 19 patients (9%) underwent palliative treatment. The median number of treatment fractions was 25 (IQR, 18–31). Concomitant chemotherapy, hormone deprivation therapy, and novel targeted therapies including immune checkpoint inhibitors were applied in 23 (11%), 70 (34%), and 4 patients (2%), respectively. The majority of patients (*n* = 171; 83%) had been treated as outpatients. The median interval between radiotherapy and questionnaire completion was 23 months (IQR, 1–38 months).

**Table 1:** Baseline characteristics of study participants (*n* = 207)

	Median (IQR)	
*Age at start of radiotherapy course [years]*	64 (56–72)	
*Gender*	*n*	%
Male	102	49.3
Female	105	50.7
*Employment status at the time of radiotherapy*		
Employed (including self-employed)	73	35.3
Unemployed	28	13.5
Retired	105	50.7
Unknown	1	0.5
*Partnership at the time of radiotherapy*		
Partner	152	73.4
No partner	54	26.1
Unknown	1	0.5
*Health insurance at the time of radiotherapy*		
Public health insurance	204	98.6
Private health insurance	3	1.4
*ECOG at the time of study participation*		
ECOG 0	156	75.4
ECOG 1	37	17.9
ECOG 2–3	13	6.3
Unknown	1	0.5
*Primary cancer*		
Breast	84	40.6
Prostate	57	27.5
Head-and-neck	19	9.2
Lung	11	5.3
Rectal	9	4.3
Skin	8	3.9
Multiple myeloma/plasmacytoma	4	1.9
Sarcoma	3	1.4
Brain	3	1.4
Esophagus	3	1.4
Lymphoma	2	1.0
Urothel	2	1.0
Liver	1	0.5
Thymoma	1	0.5
Radiotherapy region*		
Breast including lymphatic drainage	82	39.0
Prostate including lymphatic drainage	53	25.2
Head-and-neck	25	11.9
Peripheral bones	11	5.2
Lung including mediastinum	10	4.8
Axial skeleton	9	4.3
Rectal	8	3.8
Brain	6	2.9
Bladder	2	1.0
Esophagus	2	1.0
Other^1^	2	1.0
*Tumor stage at time of radiotherapy*		
UICC/AJCC 0	9	4.3
UICC/AJCC I	80	38.6
UICC/AJCC II	38	18.4
UICC/AJCC III	38	18.4
UICC/AJCC IV	33	15.9
Other^2^	9	4.3
*Intention of radiotherapy*		
Curative	188	90.8
Palliative	19	9.2
*Time between last day of radiotherapy and study participation*		
0–6 months	67	32.4
7–12 months	15	7.2
13–18 months	20	9.7
19–24 months	16	7.7
25–36 months	33	15.9
37–48 months	20	9.7
49–60 months	11	5.3
> 60 months	25	12.1
*Concomitant systemic treatment during radiotherapy*^*#*^		
Chemotherapy	23	11.1
Hormone therapy	70	33.8
Targeted therapy including immunotherapy	4	1.9
No systemic treatment	110	53.1
*Radiotherapy-induced grade 3/4 toxicity at the time of study participation*		
No	195	5.8
Yes	12	94.2
*Tumor progression at the time of study participation*		
No	199	96.1
Yes	8	3.9
*Hospitalization during radiotherapy*		
Hospitalization	34	16.4
No hospitalization	171	82.6
Unknown	2	1.0
*Treatment fractions*	25 (18–31)	
*Median time between last fraction of radiotherapy and study participation [months]*	23 (1–38)	

ECOG, Eastern Cooperative Oncology Group; IQR, interquartile range; UICC/AJCC, Union for International Cancer Control/American Joint Committee on Cancer

*As some patients were treated at multiple sites during their last course of radiotherapy, sum of radiotherapy regions is higher than overall number of patients

^#^Patients who received a combination of hormone therapy/chemotherapy with targeted therapy including immunotherapy were included to the latter group (i.e., targeted therapy including immunotherapy)

^1^Solely inguinal lymph nodes (*n* = 1), solely axillary lymph nodes (*n* = 1)

^2^WHO grade II (*n* = 1), WHO grade IV (*n* = 2), Durie–Salmon stage I (*n* = 1), Durie–Salmon stage III (*n* = 3), Ann Arbor stage I (*n* = 2)

### Test characteristics of the German version of the DRS

DRS comprehensibility was rated as good or very good by 98% (196 of 201) of patients (Table 2). The vast majority agreed with the statement “it is clear to which decision the questions refer” {mean [standard deviation (SD)] 1.70 [0.73]} on a 5-point Likert scale ranging from 1 to 5. Twenty-nine out of 200 patients (15%) stated that answering the DRS distressed them very or quite significantly.

**Table 2:** Results of the pre-test regarding comprehensibility, explicitness and caused distress of the German version of the Decision Regret Scale

Statement	*n*	Mean	SD
The questionnaire is comprehensible	201	1.45	0.55
I found it easy to answer the questions in the questionnaire	202	1.62	0.73
It is clear to which decision the questions refer	202	1.70	0.73
The questions are difficult to answer	201	3.73	1.25
The questions cause distress for me	200	4.17	1.23

A 5-point Likert scale was used for these questions with 1 = completely agree–5 = completely disagree

SD, standard deviation

Internal consistency of the German version of the DRS as measured by Cronbach’s *α* was 0.76 (95% CI, 0.70–0.81).

### Prevalence and determinants of decision regret

**Fig. 2:**
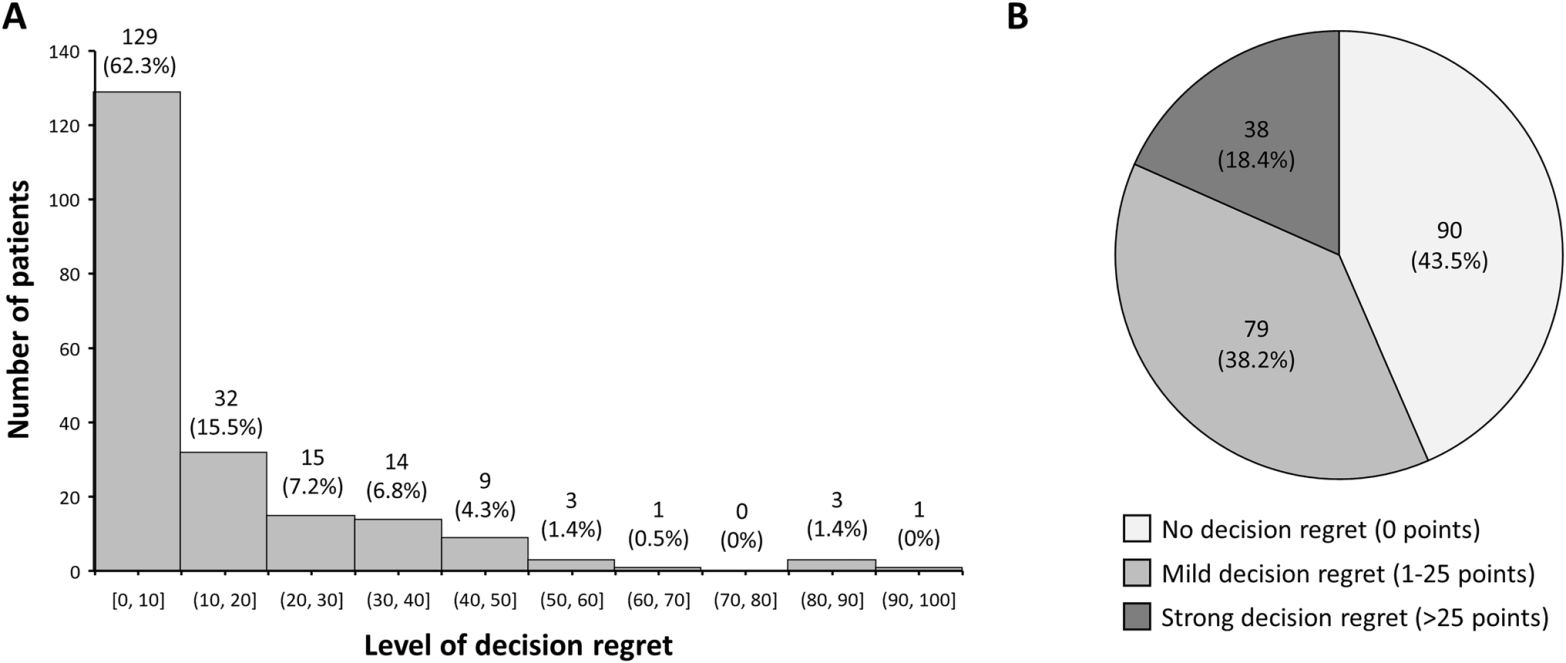
Distribution of decision regret (*n* = 207). **A** Histogram showing both absolute and relative frequencies regarding the level of decision regret, as assessed using the German version of the Decision Regret Scale. **B** Pie chart showing the frequency of absent (0 points), mild (1–25 points), and strong (> 25 points) decision regret after radiotherapy

The distribution of decision regret among cancer patients who had received radiotherapy for their disease is shown in Fig. 2. The mean and the median value of decision regret on the DRS, which ranges from 0 to 100 points, were 13.62 (SD, 18.73) and 5 points (IQR, 0–20), respectively. Decision regret was denied by 43% (*n* = 90), whereas 38% (*n* = 79) reported mild decision regret, and 18% (*n* = 38) strong decision regret.

Decision regret inversely correlated with global quality of life (Pearson’s *r* = − 0.26, *p* < 0.001), patient involvement in the decision-making process (*r* = − 0.22, *p* < 0.01), satisfaction with care (*r* = − 0.36, *p* < 0.001), social support (*r* = − 0.33, *p* < 0.001), and health literacy (*r* = − 0.29, *p* < 0.001) (Table 3). There was a positive correlation of decision regret with distress (*r* = 0.17, *p* < 0.05), depression (*r* = 0.27, *p* < 0.001), and anxiety (*r* = 0.23, *p* < 0.01).

**Table 3:** Correlation of decision regret with other patient-reported outcome measures

Variable	*r*	*p*
Global QoL	− 0.26	< 0.001
Participative decision-making	− 0.22	< 0.01
Satisfaction with care	− 0.36	< 0.001
Social support	− 0.33	< 0.001
Health literacy	− 0.29	< 0.001
Distress	0.17	< 0.05
Depression	0.27	< 0.001
Anxiety	0.23	< 0.01

Pearson correlation coefficient *r* with the according *p* value is indicated

QoL, quality of life

In the univariate analyses in which the associations of several categorical variables with decision regret were analyzed using one-way ANOVA tests, male gender (*p* < 0.05), poor ECOG performance status at the follow-up consultation (*p* < 0.001), primary cancer type (*p* < 0.001), and hospitalization during radiotherapy (*p* < 0.05) were associated with higher levels of decision regret (Table 4). In the post hoc Tukey’s test, patients with head-and-neck cancer were found to exhibit significantly higher levels of patient-reported decision regret compared to patients with breast or prostate cancer (both *p* < 0.05). Neither development of radiotherapy-induced high-grade (grade 3 or 4 according to CTCAE v5.0) toxicities (*p* = 0.125) nor tumor progression at the time of the time of study participation (*p* = 0.281) were associated with decision regret, although it should be pointed out that the number of patients with high-grade toxicities (*n* = 12) and with progressive disease (*n* = 8) were small. Among continuous variables, neither age (*r* = 0.05, *p* = 0.507) nor number of treatment fractions (*r* = 0.03, *p* = 0.707) were associated with decision regret. The time between end of radiotherapy and study participation did also not influence the extent of decision regret in our cohort (*r* = − 0.08, *p* = 0.246). In line with this, there was also no association with decision regret, if time between last day of course of radiotherapy and questionnaire completion was analyzed as an ordinal variable with 6-month and 12-month intervals (*p* = 0.443).

**Table 4:** Association of decision regret and categorical independent variables per one-way ANOVA (*n* = 207)

Variable	*n*	Mean	SD	*p*
*Gender*				
Male	102	16.81	20.76	
Female	105	10.51	16.01	**0.015**
*Employment status at the time of radiotherapy*				
Employed (incl. self-employed)	73	11.03	17.04	0.211
Unemployed	28	18.04	23.66	
Retired	105	13.51	16.80	
*Partnership at the time of radiotherapy*				
Partner	152	12.24	17.21	
No partner	54	17.75	22.20	0.063
*ECOG at the follow-up consultation*				
ECOG 0	156	10.87	16.44	
ECOG 1	37	17.52	21.41	
ECOG 2–3	13	36.54	20.55	**< 0.001**
*Primary cancer*				
Breast	84	8.63	14.63	
Prostate	57	12.37	14.15	
Head-and-neck	19	25.53	21.85	
Lung	11	11.82	14.19	
Other	36	21.48	27.01	**< 0.001**
*Intention of radiotherapy*				
Curative	188	13.09	18.96	
Palliative	19	18.86	15.74	0.201
*Time between last day of radiotherapy and study participation*				
0–6 months	67	14.93	18.33	
7–12 months	15	14.89	14.44	
13–18 months	20	11.75	14.35	
19–24 months	16	8.13	14.36	
25–36 months	33	18.79	25.59	
37–48 months	20	12.25	19.16	
49–60 months	11	15.00	25.50	
> 60 months	25	8.00	11.73	0.443
*Radiotherapy-induced grade 3/4 toxicity at the time of study participation*				
No	195	13.12	18.64	
Yes	12	21.67	19.11	0.125
*Tumor progression at the time of study participation*				
No	199	13.33	18.79	
Yes	8	20.63	16.57	0.281
*Concomitant systemic treatment*				
Chemotherapy	23	20.43	24.35	
Hormone therapy	70	9.93	16.12	
Targeted therapy incl. immunotherapy	4	15.00	17.80	
No systemic treatment	110	14.48	18.72	0.110
*Hospitalization during radiotherapy*				
Hospitalization	34	20.15	22.14	
No hospitalization	171	12.42	17.85	**0.028**

ECOG, Eastern Cooperative Oncology Group; SD, standard deviation

Bold values denote statistical significance at the *p* < 0.05 level

All parameters that were significantly associated with decision regret in the univariate analysis were simultaneously entered into a linear regression model with decision regret as dependent variable. The linear regression model significantly predicted decision regret (*F*(12,183) = 7.421, *p* < 0.001, adj. *R*^2^ = 0.283). A poorer ECOG performance status (*β* = 0.197; *p* < 0.01), lower social support (*β* = − 0.236; *p* < 0.001) and lower satisfaction with care (*β* = − 0.236; *p* < 0.001) remained independent risk factors for decision regret after radiotherapy (Table 5).

**Table 5:** Predictors of decision regret per multiple linear regression analysis

Variable	*β*	*B*	Lower 95% CI	Upper 95% CI	*p*
Gender (reference: female)	0.115	4.382	− 0.414	9.177	0.073
ECOG	0.197	6.663	1.989	11.338	**0.005**
Primary cancer (reference: other than head-and-neck cancer)	0.049	3.227	− 6.439	12.802	0.507
Hospitalization (reference: no hospitalization)	0.018	0.900	− 6.305	8.105	0.806
Quality of life	− 0.085	− 0.081	− 0.232	0.071	0.294
Participative decision-making	− 0.041	− 0.033	− 0.142	0.075	0.546
Satisfaction with care	− 0.236	− 5.939	− 9.223	− 2.655	**< 0.001**
Social support	− 0.236	− 1.166	− 1.791	− 0.540	**< 0.001**
Health literacy	− 0.083	− 3.453	− 9.462	2.557	0.258
Distress	− 0.002	− 0.016	− 1.013	0.981	0.975
Depression	− 0.013	− 0.058	− 1.087	0.971	0.911
Anxiety	0.043	0.225	− 0.878	1.327	0.688

Complete-case analysis (*n* = 196) with decision regret as dependent variable

CI, confidence interval; ECOG, Eastern Cooperative Oncology Group

Bold values denote statistical significance at the *p* < 0.05 level

## Discussion

In this single-center cross-sectional observational study performed at a large German tertiary cancer center, more than half of the investigated patients (56%) reported some form of decision regret, with 18% feeling strong decision regret after radiation treatment. Both comprehensibility and explicitness of the German version of the DRS were considered good by the participating patients, and the internal consistency as assessed with Cronbach’s α was acceptable. Higher ECOG performance status at the follow-up consultation, lower social support, and lower satisfaction with care were associated with decision regret in the multiple regression analysis.

There is a paucity of data regarding the prevalence of decision regret after radiotherapy in the German healthcare system (Köksal et al. 2023, 2022). Köksal et al. reported a strong decision regret prevalence of 13.9% after adjuvant radiotherapy in a cohort of 172 patients with breast cancer treated at a German tertiary cancer center (Köksal et al. 2023). This is well comparable to the prevalence of strong decision regret in the subgroup of breast cancer patients in our study (8 out of 84 patients [10%] with strong decision regret). In a further study, Köksal and colleagues examined the prevalence of decision regret in a group of 108 patients with head-and-neck cancer who were treated with surgery and adjuvant radiotherapy (Köksal et al. 2022). While 40.5% reported no decision regret, 30.1% indicated mild decision regret, and 29.4% even strong decision regret. Strong decision regret was reported by about half of head-and-neck cancer patients in our cross-sectional study (47%), but results should be interpreted very cautiously due to the low number of head-and-neck cancer patients in our study (*n* = 19). However, a further study also observed relatively high rates of decision regret in head-and-neck cancer patients, namely about one third of patients reporting strong decision regret at 3 and 6 months after cancer treatment (Nallani et al. 2022). The relatively high prevalence of decision regret observed in patients with head-and-neck cancer could be attributed, in part, to the fact that both definitive (chemo)radiation and surgery with risk-adapted adjuvant (chemo)radiation represent comparable treatment approaches for a significant subset of head-and-neck cancer subtypes (Henriques De Figueiredo et al. 2016; Nichols et al. 2022; Pakkanen et al. 2022; Palma et al. 2022), so that the decision-making process is stressful for the patients which increases the risk of subsequent decision regret. It has been shown that higher decisional conflict is a risk factor for later decision regret (Becerra Pérez et al. 2016), making decision support interventions such as patient-centered treatment decision aids a promising strategy to mitigate decisional conflict and subsequent regret (Bigelow et al. 2021; Stacey et al. 2017; Windon et al. 2021). In the observational study concerning decision regret in head-and-neck cancer patients from Nallani and colleagues, higher decision regret was associated with advanced disease stage at presentation, primary non-surgical treatment, and lower health literacy (Nallani et al. 2022). If further studies validate these relatively high rates of decision regret after radiotherapy in head-and-neck cancer patients, there is a need to develop multi-professional strategies to reduce decision regret in this vulnerable population.

Satisfaction with care was found to be inversely correlated with decision regret in our cohort. However, given the cross-sectional observational design of our study, it is not possible to unequivocally indicate a causal effect. While low satisfaction with radiotherapy could in theory causally contribute to higher rates of decision regret, it may also be conceivable that strong decision regret may result in higher post-hoc perceived dissatisfaction with care. A recent large multicenter study observed relatively high satisfaction with radiotherapy care in Germany (Fabian et al. 2023). Tumor entity, treatment center, and quality of life were independent determinants of patient satisfaction with radiotherapy care in this study. The fact that the treatment center itself was reported as a major determinant of patient satisfaction with radiotherapy care in the study of Fabian et al. highlights the importance of regular patient satisfaction assessment as part of the internal quality management. Indeed, regular assessments of patient satisfaction are required in the quality management guideline of the Federal Joint Committee (*Gemeinsamer Bundesausschuss*, G-BA) (Boywitt et al. 2022).

Lack of social support which was measured with the SSUK-8 was another variable that was associated with decision regret in the multiple regression analysis. In a recent population-based, prospective cohort study of 2072 patients with localized prostate cancer, social support at baseline was also found to be associated with decision regret after treatment (Wallis et al. 2022). Furthermore, a systematic review by Szproch and Maguire identified lack of social support with higher levels of decision regret after cancer treatment (Szproch and Maguire 2022). Recommending or referring patients to the psycho-oncology service as well as connecting patients with existing support groups may improve perceived social support (Korotkin et al. 2019; Sautier et al. 2014). Psychosocial care in which both the patient and his or her partner and family are addressed may also improve this outcome measure (Hartmann et al. 2010; Lorenz et al. 2019). However, especially cancer patients experiencing loneliness and social isolation remain a challenging population who suffer from low perceived social support, which is difficult to address even with psycho-oncologic interventions (Deckx et al. 2014; Hogan et al. 2002). Further prospective studies in which decision regret is assessed longitudinally are needed to further examine the relationship between social support and decision regret.

ECOG performance status at the time of questionnaire completion was found to be linked with decision regret in our study. There are various potential patient and treatment characteristics that can deteriorate patients’ ECOG performance status, e.g., higher age, presence of treatment-related adverse events, higher tumor stage, and tumor recurrence, etc. (Corrêa et al. 2012; Datta et al. 2019). As non-participants of the study exhibited a worse ECOG performance status than participants, the exact rate of decision regret may even be higher, although this remains speculative. Furthermore, it cannot be ruled out that patients at the end of their life tend to exhibit higher decision regret, as they might feel regret to have wasted their lifetime with cancer treatments that did not lead to long-term survival. Again, longitudinal analyses of decision regret including mixed-methods design and with linkage to progression-free survival are required to further elaborate on this issue and to explore reasons for decision regret after radiotherapy in more detail.

Patients who experience regret often retrospectively report being insufficiently informed (Hoffman et al. 2017; Morris et al. 2015), and patients' experiences concerning participation in the decision-making process were inversely correlated with decision regret at least in the univariate analysis of our cross-sectional study (*r* = − 0.22, *p* < 0.01). The relationship between the extent of patient participation in the decision-making process and the hazard of post-treatment decision regret is considered to be complex. While numerous studies indicate that patients actively participating in decision-making experience lower regret compared to those in passive roles (Wilding et al. 2020; Wollersheim et al. 2020), others reported opposite findings (Livaudais et al. 2013; Wagland et al. 2019). Chichua and colleagues suppose that decisional regret is linked not to the preferred or adopted role in the decision-making process, but to the discrepancy between them (Chichua et al. 2022). Both involuntary passive and active roles can result in increased regret (Mancini et al. 2012; Wagland et al. 2019). While active participation allows realistic expectations and preference expression, patients' health literacy must be considered (Joyce et al. 2020). In a study with 368 early breast cancer patients, too much perceived responsibility was associated with less baseline treatment knowledge and increased decision regret (Livaudais et al. 2013). Clinicians should therefore consider assessing patients' decisional capacity and preferences when offering recommendations and support (Chichua et al. 2022).

The participating patients found the comprehensibility of the German version of the DRS to be good, and the internal consistency, evaluated using Cronbach's *α*, was deemed acceptable. In comparison with the original DRS and the Japanese version of the DRS, internal consistency was a bit lower [Cronbach’s *α* of 0.76 in our study versus 0.81–0.92 in the original DRS (Brehaut et al. 2003) versus 0.85 in the Japanese version (Tanno et al. 2016)]. The German version of the DRS for caregivers (DRS-C), which was investigated in a cohort of caregivers of deceased people with cancer, also had a good internal consistency with a Cronbach’s *α* of 0.83 (Haun et al. 2019). Importantly, the comprehensibility of the DRS was indicated as good or very good by 98% in our cohort, supporting the usage of this questionnaire in subsequent studies.

Even though this cross-sectional study is the largest study examining the prevalence of decision regret after radiotherapy in Germany with a fairly good response rate, there are some limitations of the analysis. First, sampling error could have occurred, as the collected data originate from only a portion of the overall population who received radiotherapy in our institution. We attempted to partly address this issue by comparing the key demographic variables between participants and non-participants. Second, the missing longitudinal analysis of the degree of decision regret over time prevents an in-depth analysis about the duration of decision regret after radiotherapy. In a large longitudinal analysis about decision regret in patients with prostate cancer, the percentage of patients reporting regret increased over time in patients who were treated with radiotherapy, whereas it decreased in patients undergoing active surveillance (Hurwitz et al. 2017). Another study found relatively stable rates of decision regret over time in patients with localized breast cancer (Martinez et al. 2015). Third, given the single-center approach of our study, caution is warranted regarding the generalizability and transferability of our results to other centers in Germany. As patients with breast cancer (41%) and prostate cancer (28%) comprise the majority of patients treated with radiotherapy, our analyses regarding decision regret in patients with other cancer types are limited by the low patient number for those cancer types, so that multi-center studies with larger sample sizes are required. In this context, it has also to be mentioned that patients who had received radiotherapy with palliative intention were underrepresented in our cohort, so that transferability of our findings to this cohort is complicated. Last, recall bias may have occurred when patients were asked about their satisfaction with radiotherapy care as well as their involvement in the decision-making process, as median time between last day of radiotherapy and questionnaire completion was 23 months.

## Conclusions

Almost a fifth of cancer patients in our cohort reported strong decision regret and more than half at least some form of decision regret regarding their last course of radiotherapy. Strong decision regret was associated with a poor performance status, low social support, and dissatisfaction with care. The German version of the DRS was found well understandable, supporting its usage in further studies. Additional research is needed to investigate risk factors for decision regret across various types of cancer.

## Supplementary Information

Below is the link to the electronic supplementary material.

Supplementary file1 (PDF 503 KB)

## Data Availability

The data that support the findings of this study are available from the corresponding author upon reasonable request.
