# Supplementary material for: Decision regret of cancer patients after radiotherapy: results from a cross-sectional observational study at a large tertiary cancer center in Germany
Source: J Cancer Res Clin Oncol. 2024 Mar 28;150(3):167. doi: 10.1007/s00432-024-05638-0 (PMC10978708; doi:10.1007/s00432-024-05638-0)

Supplementary table 1: Comparison of patient characteristics between study participants and non-participants. ECOG, Eastern Cooperative Oncology Group; IQR, interquartile range.

		Participants		Non-participants		
		**Median (IQR)**		**Median (IQR)**		***p***
**Age [years]**		64 (56-72)		68 (58.75-75)		0.092[a]
		**n**	**%**	**n**	**%**	
**Gender**	Male	102	49	46	43	
	Female	105	51	62	57	0.259[b]
**ECOG at follow-up consultation**	ECOG 0	156	75	57	53	
	ECOG 1	37	18	27	25	
	ECOG 2-4	12	6	20	19	
	Unknown	1	1	4	4	**<0.001[b]**
**Primary cancer**	Breast	84	41	45	42	
	Prostate	57	28	23	21	
	Head-and-neck	19	9	10	9	
	Lung	11	5	7	6	
	Rectal	9	4	3	3	
	Skin	8	4	3	3	
	Multiple myeloma/plasmacytoma	4	2	0	0	
	Sarcoma	3	1	1	1	
	Brain	3	1	4	4	
	Esophagus	3	1	2	2	
	Lymphoma	2	1	2	2	
	Urothel	2	1	4	4	
	Other	2	1	4	4	0.451[b]

[a]unpaired t-test
[b]χ^2-test

Extract from the questionnaire in which the German version of the Decision Regret Scale is shown. Decisions refer to the last course of radiotherapy which was actively communicated to the participants.

	Ich stimme zu	Ich stimme über-wiegend zu	Weder noch	Ich stimme über-wiegend nicht zu	Ich stimme nicht zu
Die Entscheidungen waren richtig.	❍1	❍2	❍3	❍4	❍5
Ich bereue die getroffenen Entscheidungen.	❍1	❍2	❍3	❍4	❍5
Ich würde die gleiche Wahl treffen, wenn ich mich nochmals entscheiden müsste.	❍1	❍2	❍3	❍4	❍5
Die Entscheidungen haben mich sehr beeinträchtigt	❍1	❍2	❍3	❍4	❍5
Die Entscheidungen waren klug	❍1	❍2	❍3	❍4	❍5

Five questions (in German) regarding the comprehensibility and explicitness of the German version of the Decision Regret Scale, each with a 5-point Likert scale.

Bitte beurteilen Sie die Verständlichkeit des Fragebogens zum Entscheidungsbedauern:

1. Der Fragebogen ist verständlich.	1<br>Ich stimme voll und ganz zu	2<br>Ich stimme eher zu	3<br>Weder noch	4<br>Ich stimme eher nicht zu	5<br>Ich stimme überhaupt nicht zu
2. Es fiel mir leicht, die Fragen im Fragebogen zu beantworten.	1<br>Ich stimme voll und ganz zu	2<br>Ich stimme eher zu	3<br>Weder noch	4<br>Ich stimme eher nicht zu	5<br>Ich stimme überhaupt nicht zu
3. Es ist eindeutig, auf welche Entscheidung die Fragen sich beziehen.	1<br>Ich stimme voll und ganz zu	2<br>Ich stimme eher zu	3<br>Weder noch	4<br>Ich stimme eher nicht zu	5<br>Ich stimme überhaupt nicht zu
4. Die Fragen sind schwierig zu beantworten	1<br>Ich stimme voll und ganz zu	2<br>Ich stimme eher zu	3<br>Weder noch	4<br>Ich stimme eher nicht zu	5<br>Ich stimme überhaupt nicht zu
5. Die Fragen belasten mich.	1<br>Ich stimme voll und ganz zu	2<br>Ich stimme eher zu	3<br>Weder noch	4<br>Ich stimme eher nicht zu	5<br>Ich stimme überhaupt nicht zu

Details of the multiple linear regression analysis

Predictors of decision regret per multiple linear regression analysis. Complete-case analysis (n=196) with decision regret as dependent variable. The variance inflation factor (VIF) as a measure of multicollinearity is also presented. CI, confidence interval; ECOG, Eastern Cooperative Oncology Group.

Variable	β	B	Lower 95% CI	Upper 95% CI	*p*	VIF
**Gender (reference: female)**	0.115	4.382	-0.414	9.177	0.073	1.114
**ECOG**	0.197	6.663	1.989	11.338	**0.005**	1.339
**Primary cancer (reference: other than head-and-neck cancer)**	0.049	3.227	-6.439	12.802	0.507	1.482
**Hospitalization (reference: no hospitalization)**	0.018	0.900	-6.305	8.105	0.806	1.409
**Quality of life**	-0.085	-0.081	-0.232	0.071	0.294	1.778
**Participative decision-making**	-0.041	-0.033	-0.142	0.075	0.546	1.256
**Satisfaction with care**	-0.236	-5.939	-9.223	-2.655	**<0.001**	1.195
**Social support**	-0.236	-1.166	-1.791	-0.540	**<0.001**	1.123
**Health literacy**	-0.083	-3.453	-9.462	2.557	0.258	1.470
**Distress**	-0.002	-0.016	-1.013	0.981	0.975	1.539
**Depression**	-0.013	-0.058	-1.087	0.971	0.911	3.929
**Anxiety**	0.043	0.225	-0.878	1.327	0.688	3.085

Model summary of the linear regression model.

	Value
**R^2**	0.327
**Adj. R^2**	0.283
**Durbin-Watson-Statistics**	1.889

ANOVA of the regression model.

	Sum of Squares	df	Mean Square	F	*p*
**Regression**	23138.254	12	1928.188	7.421	<0.001
**Residual**	47547.162	183	259.821		
**Total**	70685.417	195			

Cook's Distance as a measurement of influential outliers within the predictor variables.

	Minimum	Maximum	Mean	Standard Deviation	N
**Cook's Distance**	0.000	0.128	0.007	0.016	196

Histogram of regression standardized residuals to evaluate normal distribution of regression standardized residuals

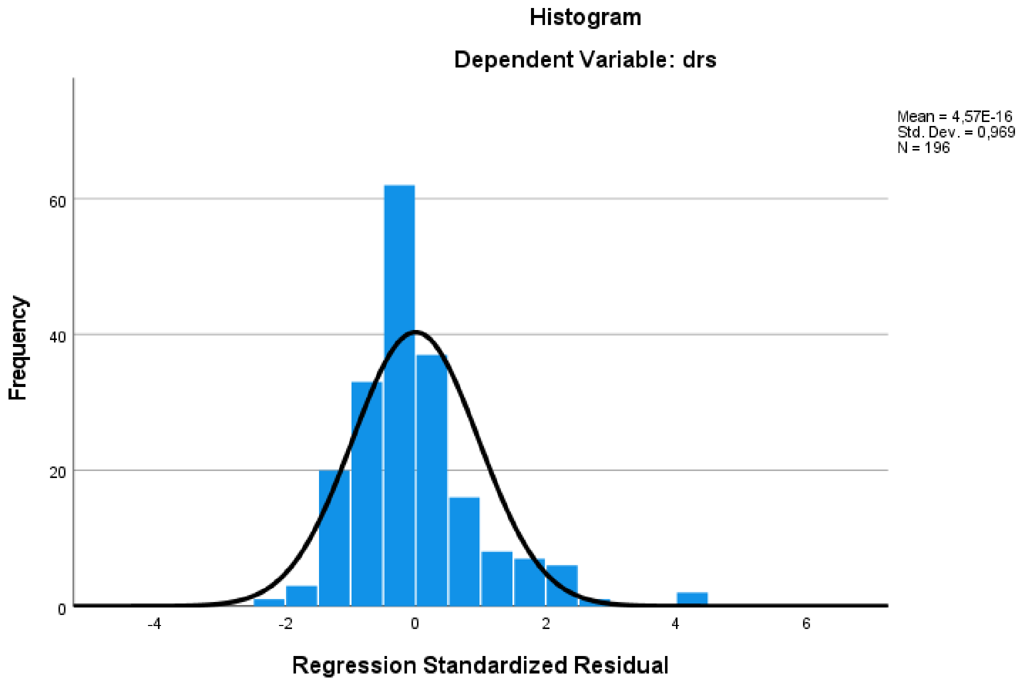

Predicted Probability plot in order to check normality

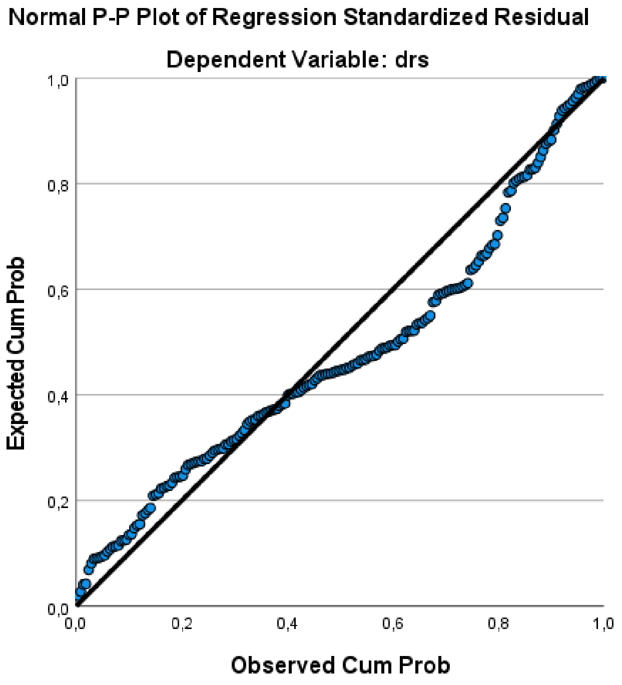

Scatterplot of the regression standardized residuals and standardized predicted values (as a measurement for homoscedasticity).

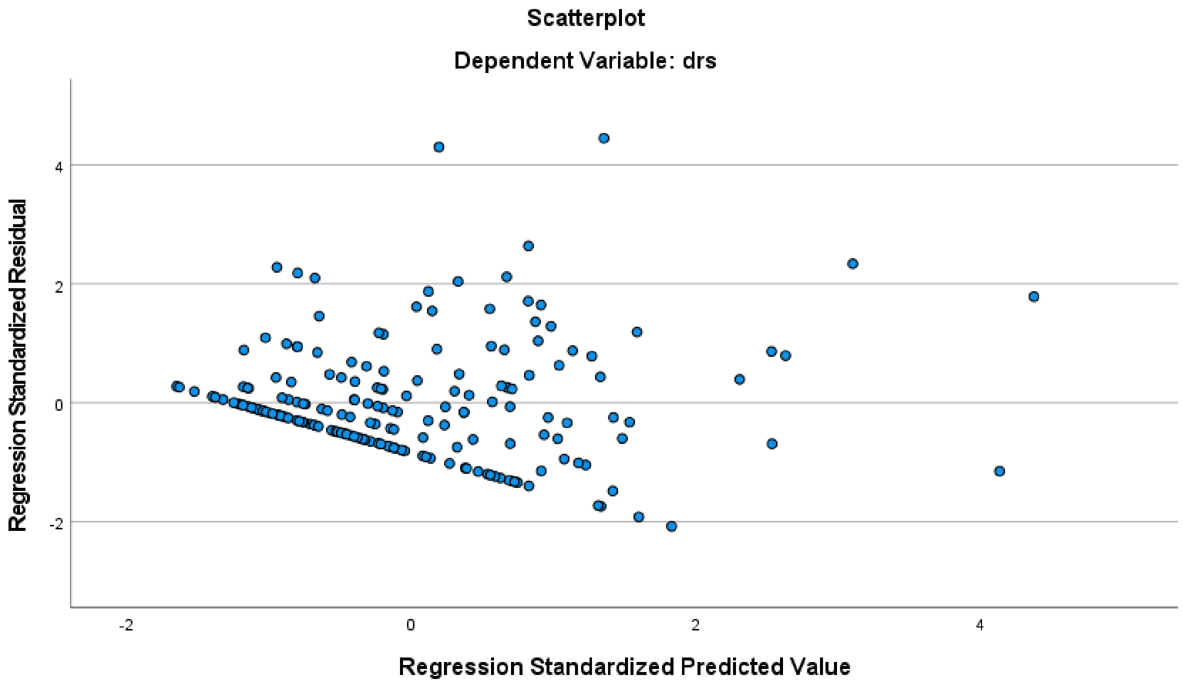

Supplement: Supplementary file 1 — Supplementary file1 (PDF 503 KB) [file 432_2024_5638_MOESM1_ESM.pdf]
